# Use of the creative arts in psychotherapy with people diagnosed with psychotic disorders and in teaching trainees about the experience of psychosis

**DOI:** 10.1192/j.eurpsy.2026.11726

**Published:** 2026-07-31

**Authors:** L. Mehl-Madrona, B. J. Mainguy

**Affiliations:** 1 Research, Coyote Institute, Orono; 2 Psychiatry, Northern Light Acadia Hospital, Bangor; 3 Education, Coyote Institute, Orono, United States

## Abstract

**Introduction:**

Creative arts therapies, particularly drama therapy techniques, are useful in psychotherapy

**Objectives:**

We aim to show how the creative arts can be used in the treatment of psychosis and in training people about the experience of psychosis.

**Methods:**

The techniques can be used in one-on-one appointments or in groups. Useful approaches include the client’s making figures to represent their voices or visions. With the help of the therapist, they interact with the figure to reduce its fearfulness and to aid them to gain mastery over the voice or vision. Drawings are also useful. In a group context, each voice can be represented by a mask (sometimes including a costume) and the voices and their interactions with the person who has them can be enacted by the group. Trainees who participate in these enactments gain first-hand experience of the experience of psychosis. Voices can be put to puppets, to stuffed animals, and to other objects to help people manage their voices better. Student participation in this process leads to an improved understanding of the psychotic experience, and improved conceptualization for how to be helpful therapeutically to people diagnosed with psychosis. In a case series of 12 clients, working with creative arts methods in one-on-one appointments was associated with substantial improvement in their psychotic symptoms. In a case series of six groups, these methods were used. We present students’ and patients’ observations on their participation in these therapies.

**Results:**

Similarities observed included initial reluctance from clients to engage in this process. Some coaching is required which is therapeutic in itself. Reasons for reluctance included the belief that the voice/being could harm them or take control of them and make them commit acts against their will. Narrative therapy techniques exist for challenging these beliefs and demonstrating the lack of power of voices/beings. Once a safe space was created, in one-on-one sessions, the therapist played the being, but behind a mask or a figure to form a clear boundary between the voice and the therapist. Interactions with the voice/being were rehearsed until the client was no longer afraid of the voice. A story was created about the origins of the voice/being and where it could go when it left the client. Group therapies included enactment of ineraction with the voice/being using all group members. All clients who were willing to work with this process for at least 8 sessions reported improvement and, over the course of 6 months, significantly reduced their dose of antipsychotic medication.

**Conclusions:**

We observe that enactment with objects in the physical world seems to bind the terror and anxiety often associated with psychosis in such a way as to reduce it to levels that can be managed in psychotherapy.

**Disclosure of Interest:**

None Declared

